# Enhanced Visualisation of Colorectal Tumours via Topical Application of EMI-137 in a Methylcellulose-Based Formulation: An ex vivo Feasibility Study

**DOI:** 10.1007/s11307-025-02042-z

**Published:** 2025-08-18

**Authors:** Elham Zonoobi, Daan G. J. Linders, Stefan Harmsen, María Rita Rodríguez Luna, Shadhvi S. Bhairosingh, Dima D. A. Almandawi, Ronald L. P. Van Vlierberghe, Marvin W. J. Nogaitzig, Christophe Portal, Stijn A. L. P. Crobach, Michele Diana, Gilbert Noordam, Davey van den Burg, Elke E. M. Peters, Andreas W. K. S. Marinelli, Rob A. E. M. Tollenaar, Denise E. Hilling, Peter J. K. Kuppen, Alexander L. Vahrmeijer

**Affiliations:** 1 Department of Surgery, Leiden University Medical Center, Albinusdreef 2, 2333 ZA Leiden, The Netherlands; 2 Hospital de Barcelona, Av. Diagonal, 660, Les Corts, 08034 Barcelona, Spain; 3 ICube Lab, Photonics Instrumentation for Health, 300 Bd Sébastien Brant, 67400 Illkirch-Graffenstaden, France; 4 Edinburgh Molecular Imaging Limited. Nine Edinburgh Bioquarter, 9 Little France Road, Edinburgh, EH16 4UX UK; 5 Department of Pathology, Leiden University Medical Center, Albinusdreef 2, 2333 ZA Leiden, The Netherlands; 6 Department of Surgery, University Hospital of Geneva, Rue Gabrielle-Perret-Gentil 4, 1205 Geneva, Switzerland; 7 Departement of Pathology, Haaglanden Medisch Centrum, The Hague, 2512 VA The Netherlands; 8 Department of Surgery, Haaglanden Medisch Centrum, The Hague, 2512 VA The Netherlands; 9 Department of Surgical Oncology and Gastrointestinal Surgery, Erasmus MC Cancer Institute, University Medical Center Rotterdam, Rotterdam, 3015 GD The Netherlands

**Keywords:** Molecular imaging, Topical application, Formulations, Fluorescence, c-Met receptor, Colorectal cancer

## Abstract

**Background:**

Fluorescence-guided molecular imaging may improve colorectal cancer (CRC) patient outcomes by enabling early detection and better surgical treatment, relying on developing targeted fluorescent tracers to highlight tumours. This study investigates visualising primary colon tumours by topically applying EMI-137, a targeted fluorescent tracer designed to bind to c-Met receptor. We introduce a novel viscous formulation to enhance the tracer's performance, aiming for a clear, robust fluorescent signal by improving contact with mucosal surface of *ex vivo* colon specimens.

**Methods:**

We evaluated fluorescence properties of EMI-137 in phosphate-buffered saline (PBS) and in methylcellulose (m-cellulose) and determined emission spectrum of the tracer in both formulations. Flow cytometry was used to determine EMI-137's specificity for c-Met receptor and its optimal concentration. Live-cell imaging visually confirmed EMI-137's fluorescence signal for the c-Met receptor, highlighting its distinctive characteristics across various solvents. In a prospective cohort study, freshly excised colon cancer specimens were incubated with EMI-137 in PBS or m-cellulose. Specimens underwent a meticulous washing process. Near-infrared fluorescence imaging was performed and compared with histopathological analysis to validate detection accuracy.

**Results:**

Fluorospectrometry showed that m-cellulose enhanced EMI-137's fluorescence intensity compared to PBS. Flow cytometry showed dose-dependent binding of EMI-137 in HT-29 cells, with an optimum at 500 nM. Microscopy confirmed targeting of c-Met receptors. Topical EMI-137 dissolved in m-cellulose visualised colon tumours effectively, resulting in a high tumour-to-background ratio. Histopathological analysis confirmed c-Met expression in these colon tumours.

**Conclusion:**

EMI-137 in a novel viscous vehicle effectively imaged c-Met expressing colon tumors, potentially facilitating fluorescent-guided tumor imaging.

**Graphical Abstract:**

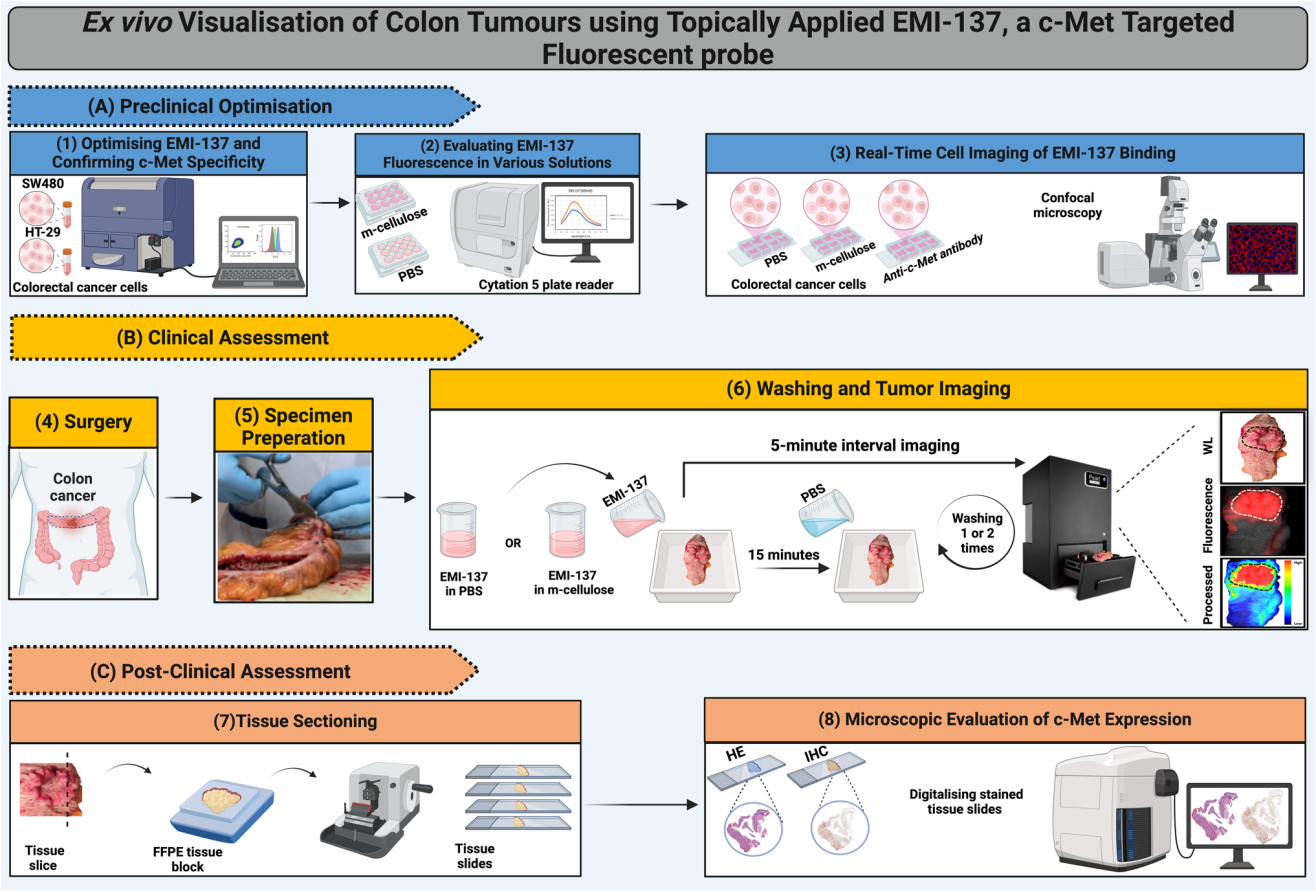

**Supplementary Information:**

The online version contains supplementary material available at 10.1007/s11307-025-02042-z.

## Introduction

Colorectal cancer (CRC) ranks as one of the most frequently diagnosed malignancies in the Western world and stands as the third leading cause of cancer-related mortality [1, 2]. It is well accepted that most CRCs slowly develop from precursor lesions such as adenomatous polyps via the adenoma-carcinoma sequence. Endoscopic screening and precursor lesion removal are common in CRC prevention but have a high miss rate of nearly 30% [2, 3]. This relatively high miss rate is due to factors like subtle morphological features (e.g. small size, less than 10 mm; or flat shape) and challenging locations (e.g., ascending colon) as well as poor bowel preparation and interobserver variability [4]. The established standard of care for treating diagnosed CRC remains surgery, which aims at complete tumour removal to enhance disease-free survival and lower cancer recurrence risks. Despite advancements in surgical techniques and use of preoperative imaging (i.e. CT, MRI, and PET scans), surgeons still mostly depend on visual inspection and palpation, along with the preoperative endoscopic application of a blue dye (ink) to the tumor site. Consequently, achieving tumour-free margins continues to be challenging in locally advanced rectal cancer cases even with the availability of advanced medical technologies such as robotic surgery, laparoscopic surgery, and the Transanal Total Mesorectal Excision (TaTME) approach [5]. Thus, there is a clear need for the development of novel techniques that can improve the detection of CRC and its precursors during screening endoscopy as well as intraoperative tumour visualisation to achieve more adequate tumour resections, particularly for locally advanced rectal cancer cases where achieving free margins can be challenging [3, 6–8]. Molecular imaging using near-infrared fluorescent (NIRF)-labelled probes with high affinity to overexpressed receptors found on neoplastic cells has shown promise to enable more accurate tumour visualisation.

Upregulation of c-Met has been identified as an early indicator in the transition of CRC from adenoma to metastatic stages, making c-Met a promising target for tumour targeting [9, 10]. EMI-137 (Edinburgh Molecular Imaging Ltd, Edinburgh EH16 4UX, United Kingdom), a fluorescent tracer designed to target the extracellular domain of c-Met and has absorption peak at 653 nm and emission peak at 675 nm [11] showed effectiveness in guiding resection of colorectal polyps [11], colonic tumours [12], Barret's neoplasia [13], thyroid [14] and penile cancer [15]. In a recent study, EMI-137 was used to detect colonic cancer during laparoscopic colorectal resection in 9 patients in which 44% showed mild fluorescence after 1–3 h after intravenous (IV) administration [12].

In addition to topical use, EMI-137 has also been studied for systemic administration during colorectal surgery, as reported by Armstrong et al., where it demonstrated the feasibility of intravenous application to support fluorescence-guided laparoscopic-assisted tumour resection [12]. Furthermore, intravenous EMI-137 has proven to be safe and useful, serving as a “red flag” in detecting dysplastic polyps during fluorescence-guided colonoscopy [4, 11].

Although IV administration of EMI-137 demonstrated good results in detecting colorectal tumours the above indications, its topical application, which could potentially lead to quicker clinical implementation, has not yet been assessed. Compared to IV administration, topical application methods, such as spraying, offer the advantage of on-the-spot decision-making when a suspicious lesion is encountered during colonoscopic screening. Furthermore, unlike IV administration, higher concentrations of tracers may be achieved locally at the mucosal surface. This approach potentially allows for direct targeting, rapid contrast enhancement, and a significant reduction in systemic exposure and potential risks of associated toxicities.

The clinical translation of EMI-137 as a topical agent in CRC screening and prevention relies on its efficient distribution across the mucosal layer following topical administration. However, achieving complete mucosal coverage remains a challenge, potentially compromising the diagnostic utility of this imaging agent. In prior work from our group, we demonstrated that without adequate formulation, topical tracers may suffer from poor mucosal adhesion, dripping, or pooling, which limit clinical effectiveness [16]. To optimise the benefits of EMI-137 as a topical agent, our research has focused on developing a viscous formulation that improves mucosal coverage and adhesion to enhance tumour exposure to EMI-137. In the current study, methylcellulose (m-cellulose), which has previously been utilised in various biomedical applications for its biocompatibility [16, 17], was used as an excipient to enhance the adhesion and distribution of EMI-137 following topical administration. Here, we show that a formulation of EMI-137 using m-cellulose enabled accurate tumour delineation following topical administration on freshly resected human colorectal specimens in an *ex vivo* imaging setting. In this study, we used post-operative colon tumour specimens to test the feasibility of detecting colorectal tumour tissue by topical application of EMI-137.

## Materials and Methods

### Materials

All chemicals were obtained from Sigma Aldrich at the highest purity available unless stated otherwise. The antibodies used included anti-Hu c-Met from eBioscience™, Goat anti-rat-Alexa 647 from Invitrogen, and anti-c-Met from Abcam.

### Cell Line Culture

Human CRC cell lines, HT-29, characterised by high c-Met expression [18], and SW-480, exhibiting minimal c-Met expression [4], were obtained from ATCC. Cells were grown in RPMI1640 medium supplemented with L-Fglutamine (Gibco, Invitrogen, Carlsbad, CA, USA), 10% FBS (Hyclone, Thermo Fisher Scientific), and antibiotics (penicillin/streptomycin, 100 IU/ml each; Invitrogen).

### M-Cellulose Preparation

25 ml PBS were heated to 56 °C, and 0.6 g of m-cellulose was added, stirring for 1 h at 56 °C. Then, 25 ml room-temperature PBS were added, stirring overnight at 4 °C. The solution was centrifuged for 3 h at 3330 g, and the top 90% was collected and stored at 4 °C.

### Tracer Preparation

Fluorescent tracer EMI-137 (a c-Met-targeting peptide conjugated to Cy5) was freshly prepared in both PBS and m-cellulose at a concentration of 500 nM on the day of the experiment, with a consistent sample volume of 50 mL for each preparation.

### Cell-Free EMI-137 Fluorescence Assays

The EMI-137 tracer was formulated in PBS and m-cellulose containing different concentrations (i.e., 200, 400, and 800 nM). A Cytation 5 plate reader (Agilent BioTek Instruments, Inc., Winooski, VT, USA) was used to quantify the fluorescence intensity of the probe in each formulation. The plate reader was set to a fixed excitation wavelength of 633 nm with emission detection ranging from 650 to 720 nm in 5 nm increments.

### Flow Cytometry Assessment

To confirm EMI-137's specificity for c-Met and its optimal concentration, flow cytometry experiments were performed. c-Met positive HT-29 and c-Met negative SW-480 cells were detached, checked for viability, and resuspended in ice-cold PBS supplemented with 0.5% bovine serum albumin (PBSA) at 500,000 cells/tube. After washing, cells were incubated with EMI-137 at concentrations ranging from 0.5 to 5000 nM for 1 h. For the positive control, HT-29 cells were stained with a primary anti-c-Met antibody (0.5 mg/ml). After incubation and washing, the cells were further stained with a secondary antibody, Goat anti-rat-Alexa 647 (A21247, 2 mg/ml), for 30 min. Following additional washes, the cells were resuspended in PBSA with propidium iodide (catalogue number P4864, Sigma Chemical Co., 1.0 mg/ml) and analysed on an LSRFortessa flow cytometer (BD Biosciences, Franklin Lakes, NJ, USA), set to count 10,000 living cells/tube. All steps were conducted on ice, protected from light, to preserve the probe and cell viability. Data were analysed using FlowJoTM software (v10.8.1, BD Biosciences).

### Real-Time Cell Imaging

To assess EMI-137's binding specificity and its fluorescence in different formulations, live-cell imaging was carried out. HT-29 cells were detached, checked for viability, and seeded into Ibidi µ-slide 8-well plates at 40,000 cells/well. Once they reached 90% confluence, cells were washed with cold PBS twice for 5 min. They were then incubated with EMI-137 (500 nM, 100 µl/well) in PBS or the m-cellulose formulation (1.9 × 10^^−6^% w/v) on ice, protected from light, for 1 h. For the positive control, HT-29 cells were incubated with a primary anti-c-Met antibody (0.5 mg/ml) for 30 min. Following incubation, the cells were washed and then stained with a secondary antibody, Goat anti-rat-Alexa 647 (A21247, 2 mg/ml), for an additional 30 min. After two more PBS washes, nuclei were stained with Hoechst 33342 (1:2500, 1 mg/ml) for 30 min, followed by three PBS washes. Imaging was done on a SP5 microscope with a HCX PL APO 63.0 × 1.40 OIL lens (Leica, Eindhoven, The Netherlands) using LASAF software (Leica) at Leiden University Medical Center's Department of Molecular Cell Biology.

### Patients

Thirteen patients diagnosed with colon cancer participated in the study. Written informed consent was obtained, and the study was approved by the ethics committee at Haaglanden Medical Center (HMC), the Netherlands (code# N22.117, approved December 2022). Freshly resected colon tissue specimens for ex vivo evaluations were obtained and studied. Patients receiving any other NIRF agent perioperatively were excluded. Patient and tumour characteristics are detailed in Supplementary Table S1.

### *Ex Vivo *Tissue Incubation and Fluorescence Imaging

To investigate the efficacy of topical EMI-137 in delineating human colon tumours using different formulations, the fluorescent probe was applied topically to freshly excised colon tumour specimens. Post-surgical resection, the specimens were immediately collected and delivered to the HMC Pathology Department for evaluation under the expertise of a dedicated pathologist (E.E.M.P). Subsequent to the assessment and adhering to standard protocols, the specimens were longitudinally opened, cleaned with running tap water, and dabbed dry with gauze. Then, the freshly excised (non-fixed) tumour specimens were subjected to topical application with EMI-137. Six specimens were treated with 500 nM EMI-137 dissolved in PBS, and seven specimens were with 500 nM EMI-137 dissolved in m-cellulose (1.08% in PBS) in a volume of 50 ml per specimen and incubated for 15 min. The optimisation of the imaging interval was determined by capturing images at varying time points (5, 10, and 15 min) throughout the incubation period using the Pearl® Trilogy Imaging System (LI-COR, Lincoln, NE, USA) in the 700 nm wavelength channel. This NIR fluorescence camera system, designed as “black box”, was selected for this validation study because it ensures consistent control over variables such as ambient light, imaging angle, and distance. Following the incubation, the specimens treated with EMI-137 in PBS or m-cellulose were rinsed once or twice with PBS, respectively, to eliminate any unbound EMI-137. The specimens were then carefully dried using sterile gauze and immediately imaged after a 15-min incubation period.

### Fluorescence Imaging Analysis

NIRF images of the longitudinally opened colon resection specimen treated with EMI-137 were captured using the Pearl® Trilogy Imaging System and analysed with QuPath software (version 0.2.3, University of Edinburgh, Scotland) and MeVisLab software (version 3.4.1, MeVis Medical Solutions, Bremen, Germany), as previously described [20]. The initial NIR fluorescence images of the entire resection specimen were uploaded into QuPath. Subsequently, tumour-positive regions were manually annotated based on macroscopic evaluation. A 1-cm border around the tumour was automatically marked as the background using the expand annotation function. Additionally, all healthy mucosa in the specimen was annotated. All three annotations were imported into the MeVisLab network. The mean fluorescence intensity (MFI), defined as the total fluorescence signal divided by the number of pixels was calculated for each annotated area. The tumour-to-background ratio (TBR) was determined by dividing the MFI of the tumour area by the MFI of the background border area. Tumours with a TBR ≥ 1.5 were classified as fluorescence-positive, while those with a TBR < 1.5 were classified as fluorescence-negative. This threshold was selected based on a previously published study on fluorescence-guided imaging, where a TBR ≥ 1.5 was considered the minimal contrast needed for reliable visual discrimination of tumor from surrounding tissue [21]. It is a commonly used as cutoff in both preclinical and clinical settings to ensure clinically actionable image contrast.

### Pathological Assessment

Specimens were processed post *ex vivo* imaging using formalin-fixation and paraffin-embedding (FFPE). From each colon tumour FFPE specimen, three 4-μm sequential sections were obtained and mounted on adhesive slides (Starfrost, Waldemar Knittel Glasbearbeitungs GmbH, Braunschweig, Germany) for fluorescent scanning, c-Met immunohistochemistry (IHC), and routine haematoxylin and eosin (H&E) staining. The FFPE IHC slides were deparaffinised with xylene, rehydrated in ethanol, rinsed with demineralised water, and treated with 0.3% hydrogen peroxide (Merck Millipore, Darmstadt, Germany) to block endogenous peroxidase. Antigen retrieval was done using Target Retrieval Solution pH 8.0 in PT Link (Agilent, Santa Clara, CA, USA) at 95 °C for 10 min. After rinsing with PBS, slides were stained with primary antibodies against c-Met (SP44, 2 μg/mL, ab227637) diluted in 1% bovine serum albumin/PBS overnight at room temperature. Subsequently, sections were incubated with EnVision anti‐rabbit horseradish peroxidase secondary antibodies (Dako) for 30 min before visualisation with DAB-kit (Dako), counterstaining with Mayer's haematoxylin solution, and mounting with Pertex (Leica Microsystems, Germany). Negative controls (PBSA) and conjugate controls (i.e., secondary antibodies only) were utilised to validate staining specificity. The c-Met IHC and histological H&E slides were digitised using the 3D-Histech 250 midi scanner (3D-Histech Ltd., Hungary).

### Scoring Method

Tumour-positive regions were identified by a board-certified pathologist (E.E.M.P.) who was blinded to the fluorescent signal. The assessment of c-Met expression was conducted in a blinded manner by an experienced board-certified gastrointestinal pathologist (A.S.L.P.C.) using the H-Score method [22]. Positive cells were classified into four staining intensity levels (-, +, + +, + + +) to calculate a c-Met expression level score. This score was determined by multiplying the percentage of positive tumour area by the respective staining intensity (1(+) × % positive + 2(+ +) × % positive + 3(+ + +) × % positive), reflecting the average expression intensity throughout the entire tumour area divided by 100. The score ranges from 0 (no expression) to 3 (100% + + + expression) [22].

### Statistical Analysis

In this study, continuous variables are shown as mean (SD) and median [IQR]. Due to non-normal distributed data, Wilcoxon signed-rank test and Friedman test, respectively, were used to compare TBR between specimens treated with EMI-137 in PBS and m-cellulose formulations. The Friedman test was employed to evaluate the trend observed over time. Correlations were analysed using Spearman's correlation. Data analysis was done using SPSS Version 16.0 (Chicago, SPSS Inc.) and GraphPad Prism 8.0.1 (GraphPad Software Inc.). P < 0.05 was considered significant.

## Results

### *In Vitro* Validation of EMI-137 Specificity and Optimal Concentration for c-Met Targeting

**Fig. 1:**
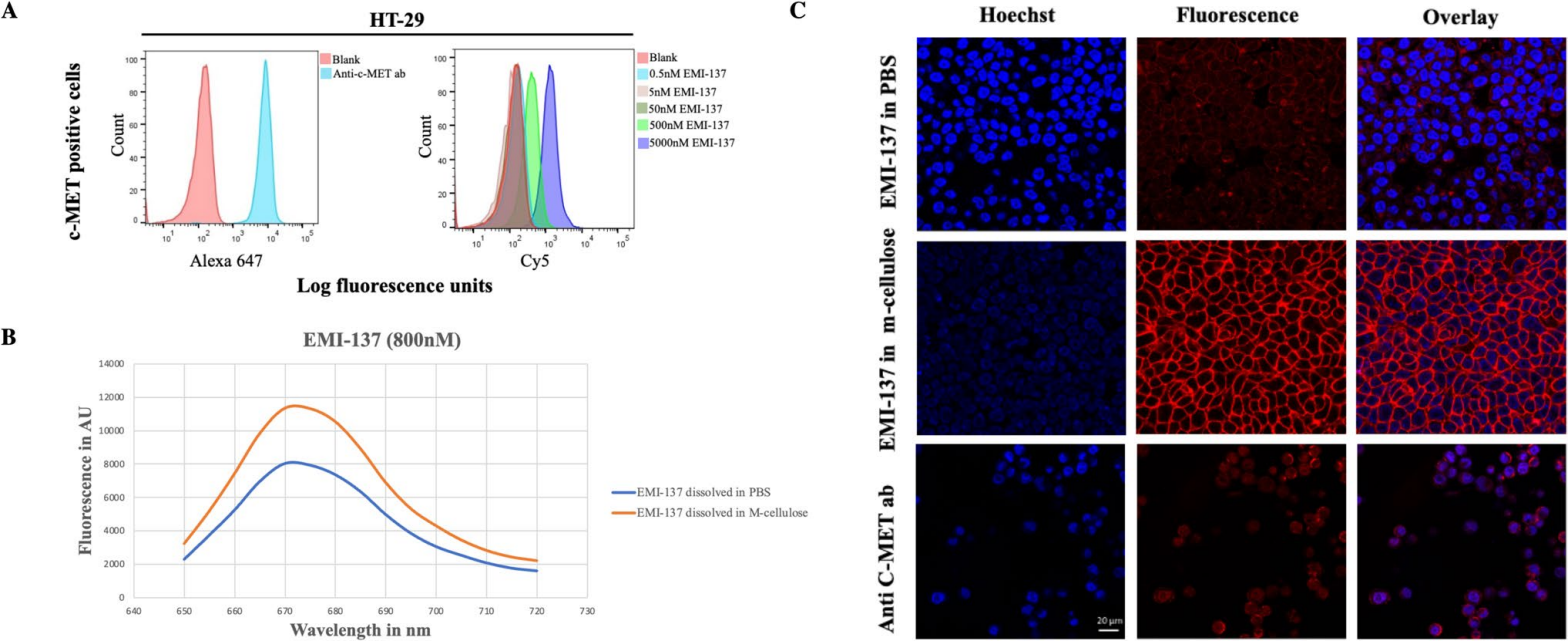
EMI-137 specificity and signal enhancement. **A** Flow cytometry was used to assess the binding of an anti-human c-Met antibody (Alexa 647 fluorescence; left) and EMI-137 at various concentrations (5000, 500, 50, 5, 0.5 nM) (Cy5 fluorescence; right) to the c-Met receptor on HT-29 cells. The optimal concentration in PBS was determined based on the probe's signal intensity**.** **B** The mean fluorescence signal intensity of EMI-137 in m-cellulose and PBS was measured using a Cytation 5 imaging plate reader in the absence of cells. The signal is represented in arbitrary units (AU), showing an increased EMI-137 emission signal intensity in m-cellulose with a marginal shift in wavelength compared to PBS. **C** Confocal microscopy images depict the specific targeting of c-Met by EMI-137 at 500 nM in both m-cellulose and PBS on HT-29 cell membranes, compared to a control anti-human c-Met antibody. The images illustrate the enhancement of fluorescence signal by m-cellulose on the cell surface. Nuclei were stained with Hoechst 33,342 (blue), and fluorescence signals from EMI-137 and Alexa 647 are shown in red, with overlaid images confirming the colocalisation of EMI-137 with c-Met on the cell membrane. For negative controls of (A), see supplementary Figure S1A, and for (C), supplementary Figure S1B

Flow cytometric analysis was performed on the c-Met-positive HT-29 cell line [18] and c-Met-negative/low SW-480 cell line [4]. After incubating the cells with different concentrations of EMI-137, the analysis revealed a dose-dependent increase in fluorescence intensity in HT-29 cells, indicating greater c-Met receptor saturation at higher concentrations (5000 > 500 > 50 > 5 > 0.5 nM, Fig. 1A) relative to SW-480 cells, which exhibited a negligible level of fluorescence even at a 5 μM concentration of EMI-137. The flow cytometry histogram for SW-480 cells is provided in Supplementary Figure S1A, confirming minimal tracer binding and supporting the specificity of EMI-137. We selected 500 nM for further studies, as it offers a strong signal while potentially reducing non-specific binding and probe consumption.

### EMI-137 Fluorescence Signal and Binding Efficiency in m-Cellulose

The fluorescence intensity of EMI-137 was measured in two formulations, PBS and m-cellulose in PBS. The results indicate that EMI-137 in m-cellulose exhibits a significantly higher fluorescence intensity compared to PBS alone at all tested concentrations (200 nM, 400 nM, and 800 nM) and across all measured emission wavelengths (λ_em_). The peak fluorescence intensity in PBS occurred around 670 nm, while in m-cellulose, a slight shift was observed around 660 nm. Despite this slight shift, EMI-137 maintained its emission wavelength profile regardless of the formulation (Fig. 1B, data for 800 nM is shown; additional concentrations are presented in Supplementary Figure S2). To explore the fluorescence signal of EMI-137 across various formulations, an *in vitro* experiment was conducted. Fluorescence microscopic images of HT-29 cells revealed that EMI-137 solubilised in m-cellulose displayed stronger and more consistent fluorescence signals across all cell membranes compared to PBS. These findings confirm that the fluorescence signal of EMI-137 in m-cellulose was clearly visible and accumulated on HT-29 cell membranes, replicating the specific binding observed with the PBS solvent. To exclude the possibility of non-specific signal retention caused by the viscous formulation, SW-480 cells (c-Met-negative) were incubated with EMI-137 in m-cellulose under the same conditions. Negligible fluorescence was observed, suggesting minimal non-specific accumulation. The corresponding fluorescence microscopy images of SW-480 cells are provided in Supplementary Figure S1B. We conclude that the enhanced signal we observe with EMI-137 in combination with m-cellulose is still c-Met-specific. This enhancement may be influenced by the increased viscosity of the m-cellulose formulation, potentially reducing non-radiative relaxation and aggregation-induced quenching. Alternatively, it may result from physicochemical interactions between EMI-137 and the polymer matrix. However, these explanations remain speculative and require further experimental validation. These hypotheses could be further investigated in future studies involving normalised fluorescence spectra and controlled comparisons across varying viscosities. These results underscore the potential of m-cellulose formulations to enhance performance in *in vitro* fluorescence-based assays (Fig. 1C). Additional fluorescence spectra at lower concentrations (200 and 400 nM) are presented in Supplementary Figure S2, confirming the concentration-independent nature of this enhancement.

### *Ex Vivo *Incubation and Fluorescence Imaging of c-Met in Freshly Resected Colon Cancer Specimens

**Fig. 2:**
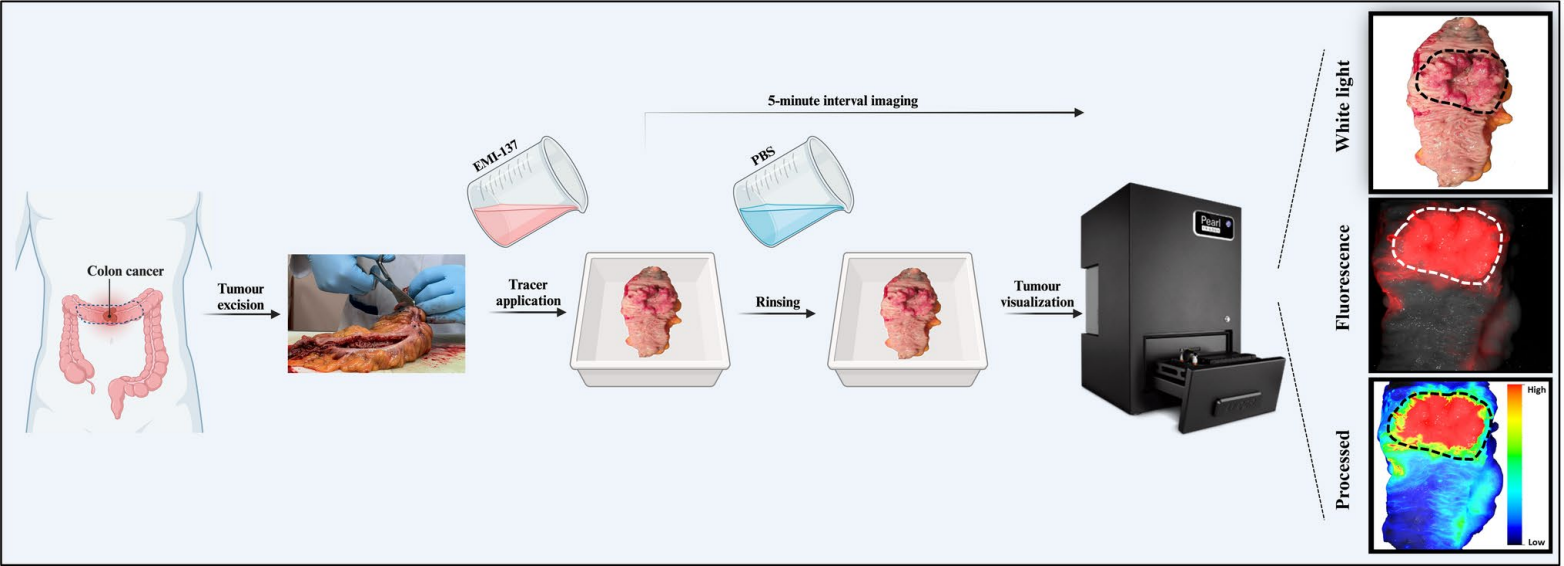
Tumour excision, incubation, and imaging workflow. The schematic overview presents the procedure from colon tumour excision to fluorescence visualisation and imaging. Post-excision, the tumour specimens were opened longitudinally and subsequently incubated in a solution with EMI-137, which consisted of either PBS or m-cellulose and imaged every 5 min during incubation using the Pearl® Trilogy Imaging System in the 700 nm wavelength channel. Following a wash to remove the unbound tracer, fluorescence imaging was again performed. Image processing of a representative sample is depicted, showing the tumour's location using white light (top) and standard imaging with a Cy-5 filter (centre). The processed image (bottom) displays the tumour-to-background ratio (TBR), indicated by the scale bar, which correlates with the fluorescence signal intensity

For the *ex vivo* examination, the study focused on assessing the feasibility of visualising primary colon tumours following topical application of EMI-137 formulated in PBS or m-cellulose containing PBS (Fig. 2). The study aimed at comparing the effectiveness of adding m-cellulose to PBS to overcome the challenges posed by gravity during topical application, which can cause the probe to drip, resulting in uneven distribution and reduced contact with the tissue. After topical administration and incubation with EMI-137 in either formulation, a fluorescence signal in tumours was detected in 12 out of 13 specimens. In all patients, the tumour was completely removed (R0). One colon tumour from the PBS-treated group was excluded from the study due to a misinterpretation of the tumour location during the handling and imaging process (Patient number 2, Supplementary Table S1).

The m-cellulose carrier used here was prepared at 1% w/v, a concentration that literature reports indicate has a moderate viscosity of ~ 100–200 centipoise (cP) at room temperature [19]. This viscosity was intentionally selected to be high enough to prevent dripping and increase tissue adherence, yet low enough to allow efficient removal of unbound tracer with standard PBS rinses. This gel-like consistency was observed to support uniform probe application without hindering rinse efficiency. To our knowledge, this study represents one of the first uses of methylcellulose for enhancing topical delivery of a fluorescence imaging agent in an oncological setting.

**Fig. 3:**
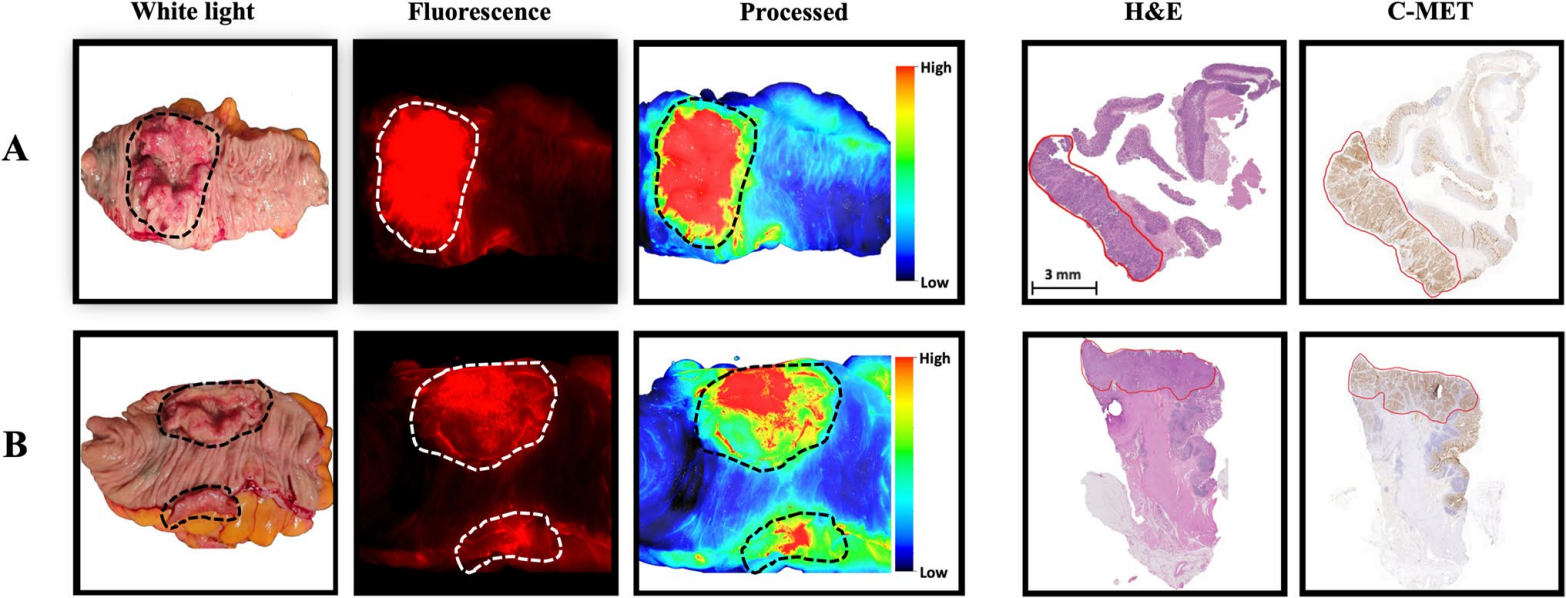
Fluorescence imaging and histological validation of c-Met targeting in tumour tissues. Overview of *ex vivo* imaging and pathological assessment for two different tissue samples incubated with EMI-137. Each panel consists of a white light image, EMI-137-based fluorescence imaging (fluorescence displayed in red), real-time image processing of the fluorescence signal (including a colour bar indicating the tumour-to-background ratio (TBR)), and corresponding heamatoxylin and eosin (H&E) staining and c-Met immunohistochemistry. **A** A tumour sample incubated with EMI-137 dissolved in m-cellulose, resulting in notably the highest tumour-to-background ratio. **B** A sample incubated with EMI-137 dissolved in PBS. In all images, the tumour area is indicated with an encircled region, facilitating the comparison between fluorescence imaging and histological analysis. All fluorescence imaging and corresponding histological assessments were performed after a 15-min incubation with EMI-137. Scale bar in histological images (H&E and IHC) = 3 mm

**Fig. 4:**
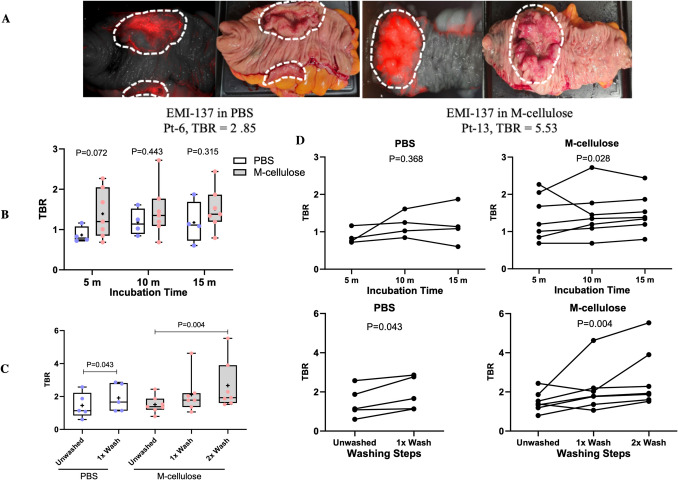
Comparative analysis of tumour-to-background ratios (TBRs) and fluorescence enhancement following topical application and washing of EMI-137. **A** Representative images of tumour specimens showing the highest EMI-137-derived fluorescence signal (red) following topical incubation and PBS washing. Images compare EMI-137 in PBS (right, Patient 6, after two washes) and in m-cellulose (left, Patient 13, after two washes). Tumour regions are outlined with white dashed lines. Note: A fluorescence colour scale was not included in panel A, as contrast was evaluated based on TBRs. **B** Tumour fluorescence intensity at 5, 10, and 15 min post-application of EMI-137 in PBS and m-cellulose. Box plots indicate minimum, Q1, median, Q3, and maximum, with the mean marked (+). **C** Tumour-to-background ratios (TBRs) across time points for both formulations. **D** Comparison of TBRs following the first and second PBS wash in both PBS- and m-cellulose-treated groups. Statistical significance was assessed using the Mann–Whitney U test (**B**), Wilcoxon signed-rank test, and Friedman test (**C**,** D**). All images were acquired using standardised exposure settings. Scale bars = 1 cm

Additionally, this study explored the impact of two different topical formulations on the fluorescent signal enhancement of EMI-137 with an emphasis on optimising incubation time and washing protocols to attain improved tumour-to-background ratios (TBRs). Our observational results demonstrated that over a 15-min incubation period, with imaging conducted every 5 min, there was an accumulation of EMI-137 in the tumour using both m-cellulose and PBS formulations compared to the surrounding normal mucosal tissues (Fig. 3). However, there was a high background due to the presence of the formulation. The median fluorescence of the tracer dissolved in m-cellulose (500 nM) was consistently higher than that of the PBS-treated group at all imaged time points (5, 10, 15 min, Fig. 4A, B). A positive trend was observed in the m-cellulose group, with a gradual increase in TBR as incubation time increased (Spearman's correlation coefficient ranging from 0.741 to 0.990, P < 0.05), especially from 5 to 15 min of incubation (Friedman test, P = 0.008). This suggests that the m-cellulose may offer advantages in sustaining prolonged identification and potentially reducing issues related to the rapid dripping or pooling of the liquid carrier. In contrast, the PBS-treated group did not exhibit a significant or consistent enhancement in TBR with extended incubation time (Fig. 4B). This could be the PBS solution draining away shortly after application from the tumour area due to its lower viscosity relative to our topical m-cellulose formulation.

Washing had a significant effect on the TBR. Following a 15-min incubation, the probe in both formulations was successfully removed using PBS washes (10 mL per wash). A single wash effectively eliminated unbound tracer, providing sufficient contrast to differentiate between tumour and normal tissue. This resulted in a maximum TBR of 2.85 with EMI-137 in PBS (Wilcoxon signed-rank test, p-value = 0.043) and of 4.6 when EMI-137 was formulated in m-cellulose (1.08% in PBS). To assess whether a second PBS wash could further improve contrast, one specimen incubated with EMI-137 in PBS was subjected to an additional rinse step, mirroring the m-cellulose protocol. The whole-specimen tumour-to-background ratio (TBR) after the first wash was 2.80 and remained unchanged at 2.80 after the second wash. This suggests that a single PBS wash was sufficient to remove unbound tracer and that the additional rinse did not enhance imaging contrast. Given the viscosity of the m-cellulose, a second wash was also performed. This additional step significantly improved the contrast between the tumour and surrounding normal tissues, leading to a substantial increase in the TBR to a maximum of 5.53 (Friedman test, p-value = 0.004). These findings indicate that optimising the application method and washing protocols, especially for the m-cellulose formulated agent, is essential for maximising TBRs and improving diagnostic yield (Fig. 4C).

### Histopathological Analysis of Surgical Tumour Specimens

Immunohistochemical (IHC) analysis demonstrated c-Met overexpression in all 13 samples (Supplementary Table S1), with the most intense expression detected at the tumour's edge (Fig. 3). Membranous c-Met expression was observed in 50%–100% of the tumour cells in each specimen (median, 93.5), exhibiting both inter- and intratumoral heterogeneity in expression level. The c-Met staining scores varied from 0.54 to 2.26, with a median of 1.5. There was no statistically significant difference in c-Met expression between tumours in the two cohorts. In both cohorts, the highest and lowest TBR values were associated with tumours exhibiting moderate c-Met expression. Variations in fluorescence image intensity might be associated with the heterogeneity of c-Met expression within the lesion.

To explore whether TBR variation correlated with c-Met expression levels, we compared the c-Met IHC scores with TBRs derived from fluorescence signal intensity after one wash in PBS, and after one and two washes in m-cellulose. A weak to moderate negative correlation was observed (Pearson r = –0.72 in PBS; –0.18 and –0.21 in m-cellulose), although this was not statistically significant due to the small sample size. These exploratory data indicate that TBR is modulated by additional factors—such as tracer distribution, wash efficiency, and local tumour architecture—beyond bulk c-Met expression alone.

## Discussion

Our study showed the potential of topical application of EMI-137 using an m-cellulose-based formulation of a c-Met-targeted NIRF tracer for the visualisation of colorectal tumours. This new innovative viscous formulation not only demonstrated effective distribution across the mucosal layer and sustained contact with the target tissue but also its capability to achieve a superior TBR within a remarkably shorter application time relative to the PBS-only control formulation.

Other studies underscore the use of topical administration of fluorescent tumour-targeting tracers in fluorescence molecular endoscopy (FME) to improve premalignant lesion detection. Nagengast et al. demonstrated that the topical application of bevacizumab-800CW significantly improved the *in vivo* TBR and increased the detection of early esophageal lesions by 33% compared to conventional white-light endoscopy [23]. Similarly, de Jongh et al. reported that FME utilising topical EMI-137 was not only safe but also effective in identifying premalignant lesions in patients with Barrett's oesophagus, although the TBRs observed were modest [13]. The topical application of EMI-137 has also been explored for surgical guidance, particularly in enhancing the identification of tumour margins in penile [15] and oral cancer [24] surgeries. Given the established suitability of c-Met as a marker for colorectal adenoma/adenocarcinoma detection [4, 11, 12, 25], and the prior validation of EMI-137 for imaging gastrointestinal (GI) malignancies [11, 12], this study aimed to post-operatively evaluate the topical application of EMI-137 for the visualisation of colorectal tumours. In addition to topical use, EMI-137 has also been studied for systemic administration before colorectal surgery, as reported by Armstrong et al., where it demonstrated the feasibility of intravenous application in combination with laparoscopic-assisted fluorescence-guided tumour resection [12].

While topical administration of targeted NIRF tracers such as EMI-137 shows promise in enhancing endoscopic and intraoperative tumour visualisation, significant challenges remain, as previously reported by others [4, 11, 13, 23]. A critical issue is the need for the probe to be distributed effectively across the mucosal layer and maintain contact with the tissue for an adequate duration to ensure a binding and accurate tumour visualisation. Factors such as dripping and pooling during endoscopy and bleeding at the excision site, can lead to redistribution of the probe and affect its accuracy [27]. Our approach successfully employed a viscous formulation to address these challenges by'anchoring'the probe to its target tissue, ensuring sufficient exposure and increased saturation of available c-Met receptors. This contrasts with earlier topical delivery methods using aqueous formulations or unmodified PBS-based solutions, which often exhibited rapid washout and limited retention time, particularly on moist or uneven mucosal surfaces [13, 27]. By introducing a viscous carrier, our approach provided improved spatial stability, consistent probe distribution, and greater fluorescence signal retention, even following washing steps.

Another critical consideration for the topical administration route is the relatively low intensity of fluorescent signals, as mentioned above. Various studies have emphasised how formulation and environmental conditions can dramatically influence fluorescence intensity and excitation/emission wavelengths, underscoring the need to carefully optimise these factors for accurate and sensitive fluorescence measurements [28–30]. For instance, the choice of formulation can influence fluorescence behaviour through aggregation-caused quenching (ACQ), which can be mitigated by increasing viscosity to restrict intramolecular motions and effectively inhibiting ACQ to boost fluorescence emission intensities [31, 32]. Cellulose derivatives, which are biocompatible and viscoelastic, have been widely used in sensing, biomedical imaging, and medical procedures [17, 33, 34]. The results reported herein introduced m-cellulose as an innovative excipient and showed it addressed the challenges associated with topical administration by counteracting gravitational forces and spatially stabilising the applied solution. Our studies, using EMI-137 tracer formulated in m-cellulose/PBS, enabled accurate tumour visualisation relative to PBS alone in both *in vitro* and *ex vivo* using freshly excised colorectal tumours. While our study focused on c-Met as the molecular target, the results of our research can be broadly applicable to various cell surface targets expressed by different tumour types. Here, we have demonstrated this viscous formulation not only prevented dripping and rapid washout but also significantly improved the tumour-associated fluorescent signal relative to normal surrounding tissues. Although we demonstrated the feasibility of the topical formulation in an *ex vivo* setting to improve tumour visualisation, clinical translation of topical application towards endo(therapeutic) and intraoperative surgical oncology scenarios could be possible.

Alternative sprayable systems, like chitosan-based mucoadhesive gels and thermosensitive hydrogels, offer strong mucosal adhesion and controlled release but may require complex preparation or temperature-controlled deliver [16, 34]. Methylcellulose was selected for this study due to its well-established biocompatibility, simple preparation, and stability at room temperature. Its reversible thermogelation properties and viscosity profile offer a practical balance between ease of delivery and effective tissue adherence in topical applications [35]. Future work could explore a head-to-head comparison with alternative gel matrices for further optimisation.

In a clinical setting, the viscous EMI-137 formulation could be delivered using standard spray catheters, gel-dispensing endoscopic nozzles, or modified irrigation devices already available in endoscopic practice. These tools would enable uniform application of the tracer to mucosal surfaces, even in anatomically complex regions. The subsequent washing steps are also feasible within current endoscopy workflows, where saline or water flushing is commonly used. Integration of a brief rinse step to remove unbound tracer would require minimal additional time. Future studies should evaluate delivery precision, mucosal clearance, and operator usability to optimise implementation during routine diagnostic or surgical procedures. However, translation to *in vivo* application must also consider physiological challenges, such as peristalsis, continuous mucus turnover, and the need for sustained tissue adherence in a live human colon. These factors could affect tracer retention and fluorescence distribution, and thus warrant further investigation in clinical trials.

Tissue penetration is a critical consideration in optimising the performance of topically applied fluorescent tracers, particularly for detecting submucosal lesions. While the maximum depth of tracer penetration during tissue incubation is not fully understood, previous studies have demonstrated effective staining and evaluation in tissues up to several cell layers deep (approximately 300 µm) [35, 36]. The superficial nature of fluorescence imaging is influenced by physical constraints; however, *in vivo* studies have shown that fluorescent tracers such as EMI-137 can be applied successfully in human tissues [13, 23]. In our study, we observed that EMI-137, when dissolved in m-cellulose, penetrated tissue within minutes. This finding highlights the potential role of solvent viscosity in influencing tracer penetration depth, an area that warrants further clinical investigation to establish a direct correlation.

While our study presents significant findings, it is not without limitations, such as the modest sample size of this initial proof-of-concept investigation. Future work will involve validating these results in a larger cohort and *in vivo* settings. Inter-patient variability in TBRs was observed, with some overlap between the PBS and m-cellulose groups. Consistent with our quantitative analysis in the Results section, there was no strong correlation between individual TBRs and c-Met IHC scores, indicating that additional factors—such as tumour morphology or local tracer distribution—contribute to signal heterogeneity. While no significant difference in c-Met expression levels was found between the two cohorts, the fluorescence heterogeneity may be attributed to spatial variations in c-Met distribution, tumour size, or mucosal surface characteristics. The observed variability underscores the complexity of translating fluorescent signal intensity into a consistent diagnostic metric and highlights the importance of correlating imaging data with underlying biological variability. Future studies will be necessary to determine whether factors such as tumour depth, location, or surface morphology contribute significantly to differences in probe accumulation and imaging performance. Although topically applied probes like EMI-137 are effective for tumour-guided imaging, combining them with systemically administered methods could improve the detection of flat cancerous lesions, enhance tracer penetration into deeper tissues, and support complete tumour removal. Additionally, formulating EMI-137 in m-cellulose may help address challenges related to assessing surgical margins after tumour resection. These potential benefits warrant further investigation in clinical studies.

The observed fluorescence-signal enhancement with EMI-137 in m-cellulose may be attributed to several factors. Increased viscosity can reduce non-radiative relaxation pathways and aggregation-induced quenching, thereby stabilising the fluorescent signal. Alternatively, physicochemical interactions between the methylcellulose polymer matrix and EMI-137 might influence the tracer’s conformation or its excited-state stability, contributing to improved emission properties. Future studies may further explore the impact of polymer characteristics and viscosity profiles for different tracers to delineate the underlying mechanisms.

This study was designed as a feasibility investigation, and therefore no formal power calculation was conducted. The sample size of 13 patients is consistent with other exploratory studies [15, 25] using fluorescence-guided imaging agents in *ex vivo* settings. Nevertheless, we acknowledge that the limited number of specimens may affect statistical power and generalisability, and future studies with larger cohorts will be needed to confirm these findings and further explore patient-level variability.

Our study indicates that the use of a viscous formulation for the topical application of NIRF agents in both *ex vivo* and *in vivo* scenarios could obviate the need for IV-administered agents in exchange for on-the-spot-decision-making for assessment and visualisation of lesions, particularly in a screening and diagnostic scenario [37]. Our approach enables more precise tumour detection during colonoscopy leading to more accurate and potentially earlier detection to improve patient outcomes.

## Conclusion

The findings of this study demonstrated c-Met as a suitable target for topically applied fluorescence-guided tumour identification. Furthermore, we introduced and showed the utility of a novel viscous formulation that improved target binding and enhanced fluorescence, resulting in more precise tumour visualisation and better patient outcomes.

## Supplementary Information

Below is the link to the electronic supplementary material.

Supplementary file1 (DOCX 4.68 MB)

Supplementary file2 (DOCX 18.2 KB)

## Data Availability

The unpublished data are available from the corresponding author upon request.
