# Supplementary material for: Enhanced Visualisation of Colorectal Tumours via Topical Application of EMI-137 in a Methylcellulose-Based Formulation: An ex vivo Feasibility Study
Source: Mol Imaging Biol. 2025 Aug 18;27(5):797–809. doi: 10.1007/s11307-025-02042-z (PMC12628436; doi:10.1007/s11307-025-02042-z)
Supplement: Supplementary file 1 — Supplementary file1 (DOCX 4.68 MB) [file 11307_2025_2042_MOESM1_ESM.docx]

Supplementary Figures

**Enhanced Visualisation of Colorectal Tumours via Topical Application of EMI-137 in a Methylcellulose-Based Formulation: An ex vivo Feasibility Study**

Original article

Elham Zonoobi ^1^, Daan G.J. Linders ^1^, Stefan Harmsen ^1^, María Rita Rodríguez Luna ^2,3^, Shadhvi S. Bhairosingh ^1^, Dima D A Almandawi ^1^, Ronald L.P. Van Vlierberghe ^1^, Marvin W.J., Nogaitzig ^1^, Christophe Portal ^4^, Stijn A.L.P. Crobach ^5^, Michele Diana ^3,6^, Gilbert Noordam ^7^, Davey van den Burg ^7^, Elke E.M. Peters^7^, Andreas W.K.S. Marinelli ^8^, Rob A.E.M. Tollenaar ^1^, Denise E. Hilling ^1,9^, Peter J.K. Kuppen ^1^, Alexander L. Vahrmeijer *^1^

1. Department of Surgery, Leiden University Medical Center, Albinusdreef 2, 2333 ZA, Leiden, The Netherlands
2. Hospital de Barcelona, Barcelona, Av. Diagonal, 660, Les Corts, 08034 Barcelona, Spain
3. ICube Lab, Photonics Instrumentation for Health, 300 Bd Sébastien Brant, 67400, Illkirch-Graffenstaden, France.
4. Edinburgh Molecular Imaging Limited. Nine Edinburgh Bioquarter, 9 Little France Road, Edinburgh, EH16 4UX, UK.
5. Department of Pathology, Leiden University Medical Center, Albinusdreef 2, 2333 ZA, Leiden, The Netherlands
6. University Hospital of Geneva, Department of Surgery, Rue Gabrielle-Perret-Gentil 4, 1205 Genève, Switzerland
7. Departement of Pathology, Haaglanden Medisch Centrum, The Hague 2512 VA, The Netherlands
8. Department of Surgery, Haaglanden Medisch Centrum, The Hague 2512 VA, The Netherlands
9. Department of Surgical Oncology and Gastrointestinal Surgery, Erasmus MC Cancer Institute, University Medical Center Rotterdam, Rotterdam 3015 GD, The Netherlands

Corresponding author:

Alexander L Vahrmeijer

Department of Surgery, Leiden University Medical Center, Albinusdreef 2, 2333 ZA, Leiden, The Netherlands.

[a.l.vahrmeijer@lumc.nl](mailto:a.l.vahrmeijer@lumc.nl)


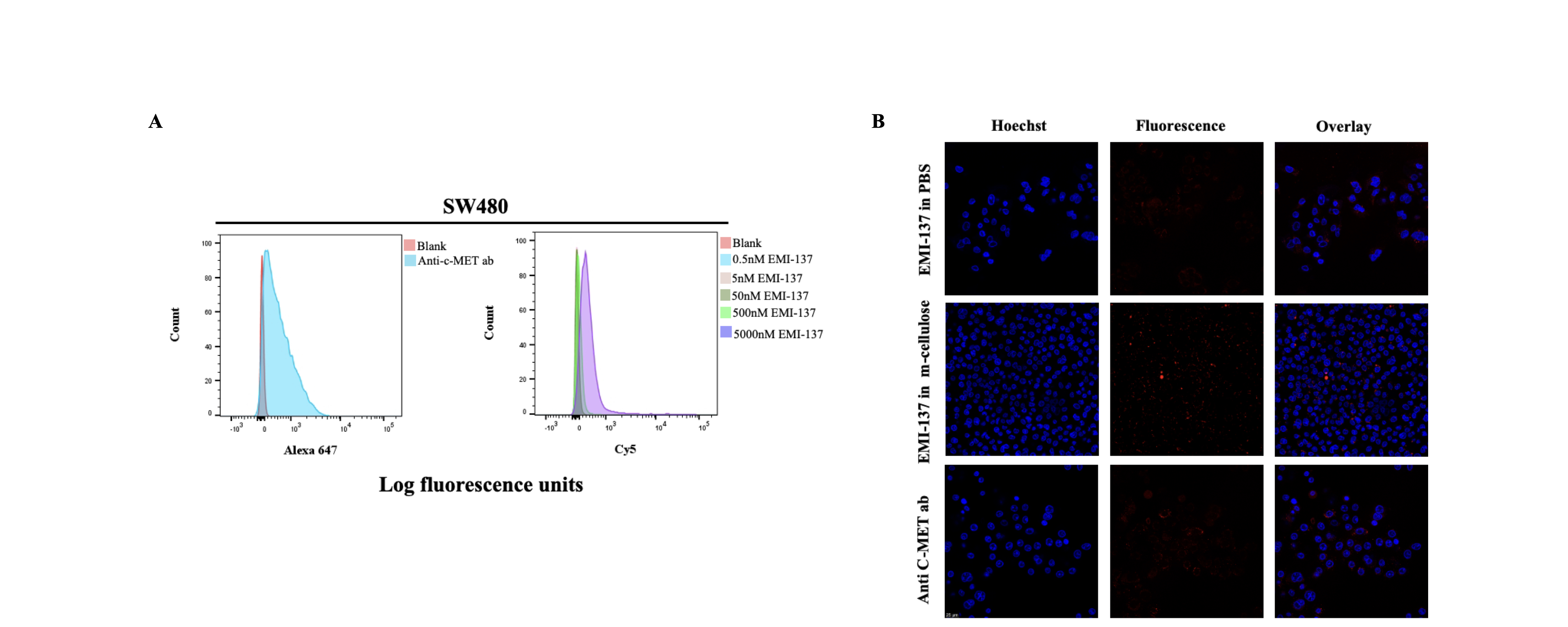


**Supplementary Figure S1. Evaluation of c-Met Expression and EMI-137 Binding in SW480 Cells (c-Met Negative Control).** This figure shows the negative controls that belong to the experiments shown in Figure 1A and 1C. **(A)** Flow cytometry histograms show c-Met expression (Alexa 647 channel) and tracer binding (Cy5 channel) in SW480 cells. The anti-human c-Met antibody confirmed negative-low c-Met expression. Binding of EMI-137 was evaluated across a concentration range (0.5 nM to 5000 nM), confirming low signal intensity at all concentrations, indicating minimal binding. **(B)** Live-cell fluorescence imaging of SW480 cells treated with EMI-137 (500 nM) in PBS or m-cellulose. Minimal fluorescence was detected in both conditions, further supporting the tracer’s specificity for c-Met. The anti-c-Met antibody control also showed very low fluorescence, as expected. Nuclei were stained with Hoechst 33342 (blue), and EMI-137 or Alexa 647 fluorescence is shown in red. Scale bar = 20 µm. Abbreviations: nM = nanomolar; ab = antibody; PBS = phosphate-buffered saline; m-cellulose = methylcellulose; µm = micrometer.


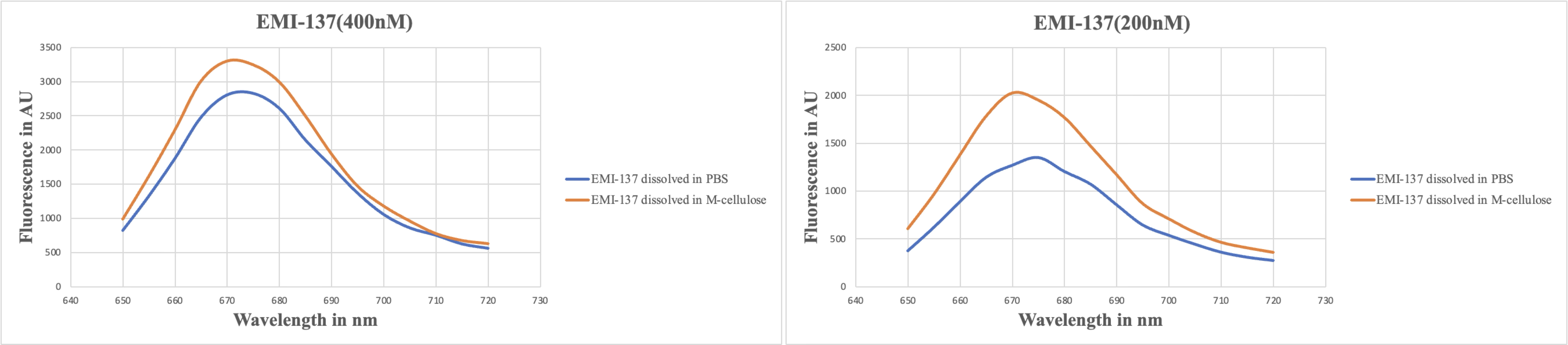


**Supplementary Figure S2. Fluorescence Emission Spectra of EMI-137 in m-cellulose and PBS at two different Concentrations.** Fluorescence intensity of EMI-137 was measured in PBS and m-cellulose at 200 nM and 400 nM using a Cytation 5 imaging plate reader (excitation at 633 nm; emission measured from 650 to 720 nm in 5 nm increments). Across both concentrations, EMI-137 consistently demonstrated a higher emission signal in m-cellulose compared to PBS. These findings show that the signal enhancement effect of m-cellulose is not concentration-dependent. Abbreviations: AU = arbitrary units; nM = nanomolar; nm = nanometer; PBS = phosphate-buffered saline; m-cellulose = methylcellulose.
