# Supplementary material for: Enhanced Visualisation of Colorectal Tumours via Topical Application of EMI-137 in a Methylcellulose-Based Formulation: An ex vivo Feasibility Study
Source: Mol Imaging Biol. 2025 Aug 18;27(5):797–809. doi: 10.1007/s11307-025-02042-z (PMC12628436; doi:10.1007/s11307-025-02042-z)
Supplement: Supplementary file 2 — Supplementary file2 (DOCX 18.2 KB) [file 11307_2025_2042_MOESM2_ESM.docx]

**Supplementary Table**

**Table S1. Patient characteristics.** This table presents the tumour characteristics of all 13 *ex vivo* patients, including clinical and pathological staging, c-Met expression, and tumour-to-background ratio (TBR) in two different cohorts: EMI-137 formulated in PBS and m-cellulose.

| **Pt. No.** | **Tumour**  **Histology** | **cT** | **cN** | **cM** | **Location** | | **pGr** | **pT** | **pN** | **pM** | **Surgery** | **Diameter**  **(mm)** | **Solvent** | | **c‐Met**  **expression**  **(%)** | **c‐Met**  **score** | **TBR**  **1x wash** | **TBR**  **2x wash** |
| --- | --- | --- | --- | --- | --- | --- | --- | --- | --- | --- | --- | --- | --- | --- | --- | --- | --- | --- |
| 1 | Adeno | - | - | - | Colon ascendens | Well/mod. diff. (low-grade) | | pT2 | N1b | N/A | HR | 35 | PBS | 85 | | 0.85 | 2,8 | 2,8 |
| 2 | Adeno | 2 | 0 | 0 | Colon ascendens | Well/mod. diff. (low-grade) | | pT2 | N0 | N/A | HR | 19 | PBS | 100 | | 1,3 | NA | N/A |
| 3 | Adeno | x | 0 | 0 | Cecum | Well/mod. diff. (low-grade) | | pT2 | N0 | N/A | HR | 15 | PBS | 91.7 | | 1.53 | 1,1 | N/A |
| 4 | Adeno | - | - | - | Sigmoid | Well/mod. diff. (low-grade) | | pT1 | N0 | N/A | SR | 30 | PBS | 98.75 | | 1.89 | 1,1 | N/A |
| 5 | Adeno | 4a | x | x | Colon ascendens | Well/mod. diff. (low-grade) | | pT2 | N0 | N/A | HR | 48 | PBS | 62 | | 0.8 | 1,7 | N/A |
| 6 | Adeno | 2 | 0 | 0 | Cecum | Well/mod. diff. (low-grade) | | pT2 | N0 | N/A | HR | 25 | PBS | 92 | | 1.48 | 2,85 | N/A |
| 7 | Adeno | 1/2 | 0 | 0 | Sigmoid | Well/mod. diff. (low-grade) | | pT3 | N1b | N/A | SR | 52 | m-cellulose | 98 | | 2.26 | 1,4 | 1,60 |
| 8 | Adeno | 2/3 | x | x | Colon ascendens | Well/mod. diff. (low-grade) | | pT3 | N1a | N/A | HR | 40 | m-cellulose | 85 | | 0.85 | 1,1 | 1,52 |
| 9 | Adeno | 3 | 0 | - | Colon ascendens | Well/mod. diff. (low-grade) | | pT4a | N2a | M1a | SR | 45 | m-cellulose | 100 | | 1.9 | 1,8 | 1,93 |
| 10 | Adeno | 3 | 0 | 0 | Sigmoid | Well/mod. diff. (low-grade) | | pT3 | N2a | N/A | HR | 50 | m-cellulose | 97.5 | | 1.45 | 2,0 | 3,90 |
| 11 | Adeno | 3 | 1 | 0 | Colon ascendens | Well/mod. diff. (low-grade) | | pT2 | N0 | N/A | HL | 40 | m-cellulose | 50 | | 0.54 | 2,2 | 2,28 |
| 12 | Adeno | 2/3 | 0 | 0 | Splenic flexure | Well/mod. diff. (low-grade) | | pT2 | N0 | N/A | SR | 50 | m-cellulose | 98.9 | | 1.9 | 1,8 | 1,87 |
| 13 | Adeno | 3b | 0 | x | Sigmoid | Well/mod. diff. (low-grade) | | pT3 | N0 | N/A | SR | 48 | m-cellulose | 95 | | 1.68 | 4,6 | 5,53 |

**Abbreviations**: Pt. No= patient number, cT = clinical T‐stage, cN = clinical N‐stage, cM = clinical M‐stage, Adeno = adenocarcinoma, pT = pathological T‐stage, pN = pathological N‐stage, pGr = pathological differentiation grade, HR= hemicolectomy right, SR = sigmoid resection , HL = hemicolectomy left, TBR = tumour-to-background ratio, N/A= not applicable, x = not staged, ‐ (a dash) = not reported, m-cellulose = methylcellulose
